# Screening anti-infectious molecules against *Mycobacterium ulcerans*: A step towards decontaminating environmental specimens

**DOI:** 10.1371/journal.pone.0231685

**Published:** 2020-08-06

**Authors:** Nassim Hammoudi, Romain Verdot, Jean Delorme, Amar Bouam, Michel Drancourt

**Affiliations:** 1 IHU Méditerranée Infection, Marseille, France; 2 Aix-Marseille University, IRD, MEPHI, IHU Méditerranée Infection, Marseille, France; 3 Assistance Publique – Hôpitaux de Marseille, Pharmacie Centrale, Marseille, France; Jamia Hamdard, INDIA

## Abstract

*Mycobacterium ulcerans*, a non-tuberculous mycobacterium responsible for Buruli ulcer, resides in poorly defined environmental niches in the vicinity of stagnant water. Very few isolates have been confirmed. With a view to culturing *M*. *ulcerans* from such contaminated environmental specimens, we tested the *in vitro* susceptibility of the *M*. *ulcerans* CU001 strain co-cultivated with XTC cells to anti-infectious molecules registered in the French pharmacopoeia. We used a standardised concentration to identify molecules that were inactive against *M*. *ulcerans* and which could be incorporated into a decontaminating solution. Of 116 tested molecules, 64 (55.1%) molecules were ineffective against M. ulcerans CU001. These included 34 (29.3%) antibiotics, 14 (12%) antivirals, eight (6.8%) antiparasitics, and eight (6.8%) antifungals. This left 52 molecules which were active against *M*. *ulcerans* CU001. Three of the inactive antimicrobial molecules (oxytetracycline, polymyxin E and voriconazole) were then selected to prepare a decontamination solution which was shown to respect *M*. *ulcerans* CU001 viability. These three antimicrobials could be incorporated into a decontamination solution to potentially isolate and culture *M*. *ulcerans* from environmental samples.

## Introduction

*Mycobacterium ulcerans* is a non-tuberculous mycobacterium responsible for Buruli ulcer, an opportunistic neglected tropical disease that also affects some non-human mammalian species [1]. *M*. *ulcerans* was first isolated in Australia in 1948 after the disease was initially described in 1897 in Uganda [2]. Phylogenetic analysis showed that *M*. *ulcerans* evolved from a common ancestor with *Mycobacterium marinum* following genomic reduction characterised by an accumulation of insertion sequences and counterbalanced by the acquisition of a giant plasmid encoding for the non-ribosomal synthesis of mycolactones, exotoxins exhibiting ulcerative, analgesic, immunosuppressive and anti-inflammatory properties [3–4]. *M*. *ulcerans* is an environmental mycobacteria, and although DNA sequences specific to *M*. *ulcerans* are routinely detected by PCR in aquatic ecosystems [5–6], its exact reservoir and routes of transmission to humans remain unknown [7]. Indeed, PCR-based data do not provide insight into the viability of these detected mycobacteria.

The first environmental *M*. *ulcerans* isolate was reported in 2008 from an aquatic insect [8], 60 years after it was first isolated from a patient [2]. The long delay between isolation from environmental sources and clinical sources illustrates the particular difficulty of isolating *M*. *ulcerans* from environmental sources, i.e., contamination by fast-growing mycobacteria, bacteria, and fungi [9–11]. An efficient decontamination protocol is key to the potential isolation of environmental *M*. *ulcerans* strains. From this perspective, different strategies have been used, including the Petroff and reversed Petroff methods, combinations of oxalic acid-NaOH, NaOH-malachite green-cycloheximide and N-acetyl-cysteine-NaOH, and Löwenstein-Jensen (LJ) medium supplemented with polymyxin B, amphotericin B, nalidixic acid, trimethoprim and azlocillin (PANTA), mycobactin J, isoniazid and ethambutol [1]. However, all these decontamination methods can adversely affect the viability of *M*. *ulcerans* [12].

To progress towards an efficient decontamination method that preserves the viability of *M*. *ulcerans*, we tested the nonantimicrobial activity of 116 antimicrobial agents, including antibiotics, antifungals, antiparasitics and antivirals listed in the French pharmacopeia, against *M*. *ulcerans* to identify molecules that could potentially be used to isolate environmental *M*. *ulcerans* strains using xenic and axenic culture media.

## Materials and methods

### Antimicrobial drugs

A collection of 116 molecules registered as antimicrobials in the French pharmacopoeia were tested for their activity against *M*. *ulcerans*. Seventy molecules were purchased from pharmaceutical companies, 45 molecules from Sigma (Lezennes, France) and one from EUROMEDEX (Souffelweyersheim, France) (S1 and S2 Tables). Each molecule was resuspended in the appropriate solvent according to the supplier’s recommendations, in order to prepare a stock solution that was aliquoted and stored at -20°C.

### Mycobacteria and cells

*M*. *ulcerans* CU001, a clinical isolate from a Ghanaian patient, was kindly provided by Professor Vincent Jarlier from the Salpêtrière hospital in Paris France [13]. It was cultured in a biosafety level 3 laboratory on Middlebrook 7H10 agar (Becton Dickinson, Le Pont de Claix, France) supplemented with 10% oleic acid, bovine albumin, dextrose and catalase enrichment (OADC, Becton Dickinson), for six weeks in an aerobic atmosphere at 30°C. Colonies were suspended in a 20-mL glass tube containing glass beads and 10 mL of Dulbecco’s phosphate buffered saline (DPBS). The suspension was vigorously vortexed and passed three times through a 26-gauge needle to separate the aggregates. The suspension was then calibrated to 10^6^ colony-forming units (CFUs)/mL corresponding to McFarland Standard no. 1. In parallel, XTC cells originating from the South African clawed toad *Xenopus laevis* [14] were grown in L15 glutaMAX medium (Gibco, ThermoFisher, Illkirch, France) supplemented with 5% heat-inactivated foetal bovine serum (FBS) and 40mL of tryptase in 75cm^2^ flasks at 28°C for seven days. Cells were detached by tapping the flasks, and 2mL of cell suspension was transferred to each well of a 12-well cell culture plate (ThermoFisher). In this study, XTC cells were used as a medium for cultivating *M*. *ulcerans* and to test the activity of molecules to mimic the biotic environment in which *M*. *ulcerans* is suspected to thrive.

### Antimicrobial assay

The 116 antimicrobials studied against *M*. *ulceran*s were assayed in a coculture system with XTC cells and *M*. *ulcerans*. In brief, each 12-well plate was divided into four columns of three wells. 20 μL of DPBS was then added to each well in column one (negative control), 20 μL of *M*. *ulcerans* CU001 suspension at 10^6^ CFUs/mL (equivalent to 0.2 X 10^5^ CFUs per well) was added to each well in column two (positive control), and 20 μL of the same *M*. *ulcerans* inoculum was added to each well in the third and fourth columns, which were supplemented with antibiotics, one antibiotic per column. The final concentration of each antibiotic used in these experiments was five times the minimum inhibitory concentration (MIC) reported in the literature. The plate was incubated at 30°C in an aerobic atmosphere for seven days, and 100 μL of each well was then plated on Middlebrook 7H10 agar plates in triplicate and incubated at 30°C for six weeks. The number of colonies (up to 150 colonies) on each plate was counted using Fiji-ImageJ software (https://imagej.net/Fiji/Downloads), and the average number of colonies grown on the three plates was calculated for each antibiotic. In this study, any molecules that left >75 colonies on plates (50%) were considered not to affect the survival of *M*. *ulcerans*.

### Trans *Mycobacterium ulcerans* (Trans MU), a decontamination medium for the recovery of *M*. *ulcerans*

**Fig 1:**
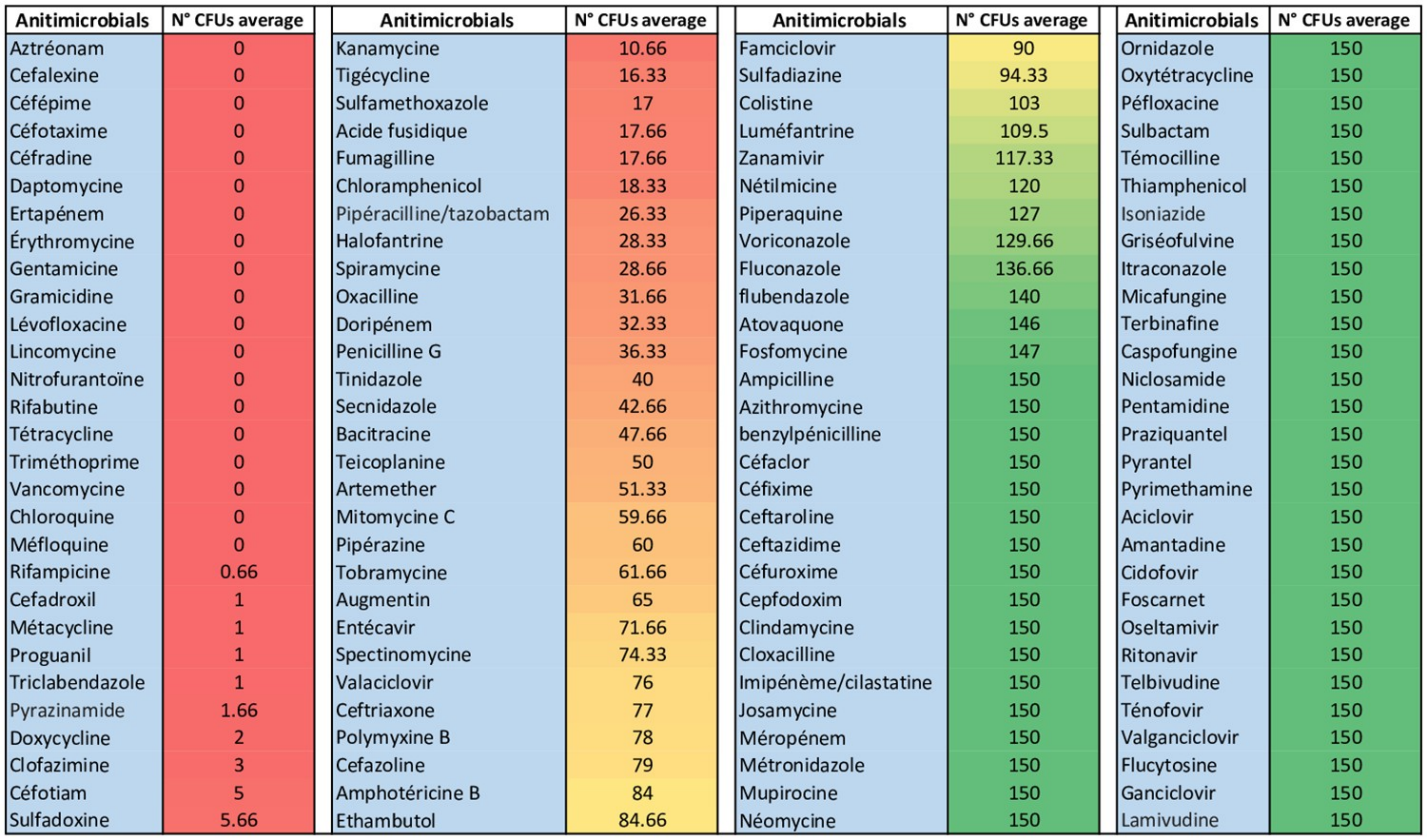
Results of the sensitivity of *M*. *ulcerans* against 116 antimicrobials, the color gradient from red to yellow includes antimicrobials that affect the viability of *M*. *ulcerans* CU001. The color gradient from yellow to green includes antimicrobials that do not affect the viability of *M*. *ulcerans*. (cut of 50% = 75 Avg CFU Nbr].

Our previous attempts to culture *M*. *ulcerans* in environmental samples were limited by three categories of contaminating organisms including fungi, Gram-negative bacteria, and Gram-positive bacteria, especially *Bacillus* (Bouam A., Hammoudi N, unpublished data). To address this contamination problem, we selected three antimicrobial agents that target these three categories, i.e., an anti-*Bacillus* (oxytetracycline), an anti-Gram-negative bacteria (polymyxin E), and an antifungal agent (voriconazole) at the same concentrations that were used in this study (Fig 1). In the first step, the antimicrobial mixture was mixed with 1% chlorhexidine (final concentration) to create a decontamination medium (Trans MUl). In the second step, the antimicrobial mixture was mixed with Middlebrook-OADC at 50°C and poured into 50–55-mm Petri dishes to create a decontamination and culture agar medium (Trans MUg). A 10^5^ CFUs/mL suspension of *M*. *ulcerans* was prepared in sterile phosphate buffered saline (PBS), which was previously used as a negative control for *M*. *ulcerans* DNA in an RT-PCR experiment. A 4-mL volume of TRANS MUl was added to 1 mL of *M*. *ulcerans* suspension and incubated for four days at room temperature with constant shaking. The mixture was then centrifuged for 15 minutes at 3,500g, and the pellet was washed and vortexed vigorously in 5 mL of a neutralising solution (composed of 200 mL of PBS, 0.6g of egg-lecithin and 2 mL of Tween 80) [15]. The suspension was centrifuged at 3,500g for 15 minutes, and the pellet was resuspended in 1 mL of sterile PBS. A 200μL volume of sample was inoculated in triplicate on Trans MUg at 30°C for two months. At the same time, a sample of sterile PBS contaminated with 10^5^ CFUs/mL *M*. *ulcerans* was directly plated on Middlebrook 7H10 agar and TRANS MUg in triplicate. A second sample, treated only with TRANS MUl, was plated on Middlebrook 7H10 agar in triplicate as a control.

## Results

### Antimicrobial assay

A total of 116 pharmaceutical molecules corresponding to four therapeutic families were tested against *M*. *ulcerans* CU001. Results revealed that 64 (55.2%) molecules did not affect the viability of *M*. *ulcerans*. These molecules included 34 (29.3%) antibiotics, 14 (12%) antivirals, eight (6.8%) antiparasitics, and eight (6.8%) antifungals, as displayed in in the yellow and green coloured cells in Fig 1, S1 Table. In contrast, 52 (44.8%) molecules altered the viability of *M*. *ulcerans*. These molecules included 42 (36.2%) antibiotics and 9 (7.6%) antiparasitics, in addition to entecavir, which yielded 71 colonies, just below the 75-colony cut-off used in this study. No other antivirals or antifungals altered the viability of *M*. *ulcerans*, as displayed in red and yellow cells in Fig 1, S1 Table.

### Trans MU as a decontamination medium

A *M*. *ulcerans* suspension in PBS was used as a positive control and yielded colonies after 30 days of incubation at 30°C. The same observation was reported for PBS contaminated with 10^5^ CFU/mL *M*. *ulcerans* cultivated on TRANS MUg, which was positive after 30 days of incubation at 30°C. In addition, Middlebrook 7H10 agar plates or TRANS MUg plates inoculated with 10^5^ CFU/mL *M*. *ulcerans* previously treated with TRANS MUl allowed *M*. *ulcerans* to grow after 30 days of incubation at 30°C.

## Discussion

We present the first, large, open-minded study of the activity of 116 molecules against *M*. *ulcerans*, the etiologic agent of Buruli ulcer [1]. The data reported in this study were validated by the positivity of positive controls, and the results reported here are in agreement with those previously published in the literature, such as the *in vitro* activity of rifampicin against *M*. *ulcerans*, which is the current basis of Buruli ulcer treatment [16, 17]. The results also concur with the *in silico* prediction of genes that confer resistance to isoniazid and pyrazinamide [18]. The data reported here were obtained by incorporating XTC cells in anti-*M*. *ulcerans* assays. The XTC cells were used as surrogates of the organic environments naturally encountered by *M*. *ulcerans* in its ecological niches, as well as in clinical tissues in which *M*. *ulcerans* behave as a pathogen.

This study broadens the spectrum of molecules active against *M*. *ulcerans in vitro*, including 20 previously reported antibiotic molecules [1] and 50 molecules encompassing not only antibiotics but also antiparasitics and one antiviral (entecavir). The MICs of these molecules are still pending determination, a task which is beyond the spectrum of the present study. Interestingly, this study reveals that subtle chemical differences, such as tetracycline oxidation to oxytetracycline, may modify the activity of molecules against *M*. *ulcerans*. Pursuing this type of observation was beyond the spectrum of this study, but the data reported here could be used for the chemical design of molecules active against *M*. *ulcerans*. This will be of interest because Buruli ulcer is classified as a neglected tropical infection [19].

The original aim of this study was to identify molecules that were inactive against *M*. *ulcerans* in such a way that they could be incorporated into decontamination solutions, while maintaining the viability of the pathogen. Indeed, it has been previously shown that storing clinical specimens in a liquid transport medium instead of storing them under dry conditions, significantly increased recovery of *M*. *ulcerans* [20]. Accordingly, we developed a liquid transport medium incorporating a cocktail of antimicrobials of interest, to remove most contaminants before sample culture on solid medium; and we are anticipating using this liquid transport medium in the perspective of isolation and culture of *M*. *ulcerans* from selected environmental samples where *M*. *ulcerans* had been detected using PCR-based methods [20].

Decontaminating or removing contaminating microorganisms from clinical and environmental samples is an essential step towards isolating *M*. *ulcerans* [9]. From this perspective, we identified 66 inactive molecules against *M*. *ulcerans in vitro*; these molecules may, therefore, be potential antimicrobials to be incorporated into culture media to decontaminate environmental and clinical samples for the isolation of *M*. *ulcerans*. Several studies have reported that delays in the transport of samples may affect the viability of *M*. *ulcerans* [21]. In addition, several decontamination methods proposed for the isolation and culture of *M*. *ulcerans*, such as the Petroff method, the reverse Petroff method, and the oxalic acid decontamination methods [22], or the use of HCl, all reduce *M*. *ulcerans* viability, resulting in culture failure [9]. Commercially available mixture PANTA (Becton Dickinson) does not modify the growth of clinical isolates of *M*. *ulcerans*, contrary to reports on *Mycobacterium leprae* [23].

In this study, we developed a protocol that allowed *M*. *ulcerans* subculture after only four days of preculture in TRANS MUl followed by 30 days of incubation on TRANS MUg at 30°C. This protocol is now used for the tentative isolation and culture of *M*. *ulcerans* from environmental samples, including in aulacode faeces samples from which *M*. *ulcerans* has been previously isolated but not subcultured [24]. Further studies may aim to compare the decontamination methods previously described for the recovery of *M*. *ulcerans* from clinical samples, with the one here developed.

## Supporting information

S1 Table Classification of the 116 antimicrobials used in this study according to their pharmaceutical class and the sensitivity of *M*. *ulcerans* CU001. (XLSX)

S2 Table Source of 116 molecules registered as antimicrobials in the French pharmacopoeia and used in these studies. (XLSX)

## References

[1] Zingue D., Bouam A., Tian RBD., Drancourt M. Buruli ulcer, a prototype for ecosystem-related infection, caused by *Mycobacterium ulcerans*. Clinical Microbiology Reviews. 2018; 31: 45–17. 10.1128/CMR.00045-17 PMC5740976 29237707

[2] MacCallum PTJC., Tolhurst JC., Buckle G., Sissons HA. A new mycobacterial infection in man. *The Journal of pathology and bacteriology*. 1948; 60: 93–122. 18876541

[3] Stinear TP, Seemann T, Pidot S, Frigui W, Reysset G, Garnier T, et al Reductive evolution and niche adaptation inferred from the genome of *Mycobacterium ulcerans*, the causative agent of Buruli ulcer. genome research. 2007; 17: 192–200. 10.1101/gr.5942807 17210928 PMC1781351

[4] Käser M, Rondini S, Naegeli M, Stinear T, Portaels F, Certa U, et al Evolution of two distinct phylogenetic lineages of the emerging human pathogen *Mycobacterium ulcerans*. BMC evolutionary biology. 2007; 7: 177 10.1186/1471-2148-7-177 17900363 PMC2098775

[5] Garchitorena A, Roche B, Kamgang R, Ossomba J, Babonneau J, Landier J, et al *Mycobacterium ulcerans* ecological dynamics and its association with freshwater ecosystems and aquatic communities: results from a 12-month environmental survey in Cameroon. PLOS Neglected Tropical Diseases. 2014; 8: 2879. 10.1371/journal.pntd.0002879 PMC4022459 24831924

[6] Roger BDT., Sébastian N., Hervé TD., Drancourt M. Detection of *Mycobacterium ulcerans* DNA in the environment, Ivory Coast. PLoS One. 2016; 11: 151567. 10.1371/journal.pone.0151567 PMC4794205 26982581

[7] Röltgen K., Pluschke G. *Mycobacterium ulcerans* disease (Buruli ulcer]: potential reservoirs and vectors. Current Clinical Microbiology Reports. 2015; 2: 35–43.

[8] Portaels F, Meyers WM, Ablordey A, Castro AG, Chemlal K, de Rijk P. et al First cultivation and characterization of *Mycobacterium ulcerans* from the environment. PLOS Neglected Tropical Diseases. 2008; 2: 178. 10.1371/journal.pntd.0000178 PMC2268003 18365032

[9] Palomino JC., Portaels F. Effects of decontamination methods and culture conditions on viability of *Mycobacterium ulcerans* in the BACTEC system. Journal of Clinical Medicine. 1998; 36: 402–408. 10.1128/jcm.36.2.402-408.1998 PMC104550 9466749

[10] Barker DJP. Buruli disease in a district of Uganda. The American Journal of Tropical Medicine and Hygiene. 1971; 74, 260–64. 5143865

[11] Barker DJP. The distribution of Buruli disease in Uganda. Trans. R. Soc. Trop. Med Hyg. 1972; 66: 867–874. 10.1016/0035-9203(72)90121-6 4653043

[12] Portaels F., Muynck A., Sylla MP. Selective isolation of mycobacteria from soil: a statistical analysis approach. Microbiology.1988; 134, 849–855. 10.1099/00221287-134-3-849 3183623

[13] Marie TR., Daniela S., Aurélie C., Vincent J., Baohong JGP. Chemotherapy-associated changes of histopathological features of *Mycobacterium ulcerans* lesions in a Buruli ulcer mouse model. Journal of Antimicrobial Chemotherapy. 2012; 56: 687–696. 10.1128/AAC.05543-11 PMC3264237 22143518

[14] Mary P., Varmac MGR., Leake J. Establishment of a cell line (XTC-2] from the South African clawed toad, Xenopus laevis. Experientia. 1973; 29: 466–467. 10.1007/BF01926785 4708349

[15] Asmar S., Drancourt M. Chlorhexidine decontamination of sputum for culturing *Mycobacterium tuberculosis*. BMC Microbiol. 2015; 15: 155 10.1186/s12866-015-0479-4 26238865 PMC4524104

[16] World Health Organization. Provisional guidance on the role of specific antibiotics in the management of Mycobacterium ulcerans disease (Buruli ulcer]. World Health Organization, Geneva, Switzerland. WHO/CDS/ CPE/GBUI/2004.10.

[17] Chauty A, Ardant MF, Adeye A, Euverte H, Guédénon A, Johnson C. et al Promising clinical efficacy of streptomycin-rifampin combination for treatment of buruli ulcer (*Mycobacterium ulcerans* disease]. J. Journal of Antimicrobial Chemotherapy. 2007; 51: 4029–4035. 10.1128/AAC.00175-07 PMC2151409 17526760

[18] Gupta SK., Drancourt M., Rolain JM. In silico prediction of antibiotic resistance in *Mycobacterium ulcerans* Agy99 through whole genome sequence analysis. The American journal of tropical medicine and hygiene. 2017; 97: 810–814. 10.4269/ajtmh.16-0478 28749770 PMC5590560

[19] Asiedu, K., Raviglione, MC., Scherpbier, R. World Health Organization, & Global Buruli Ulcer Initiative. Buruli ulcer: Mycobacterium ulcerans infection (No. WHO/CDS/CPE/GBUI/2000.1]. World Health Organization. (2000].

[20] Bratschi MW, Bolz M, Grize L, Kerber S, Minyem JC, Um Boock A, et al Primary cultivation: factors affecting contamination and *Mycobacterium ulcerans* growth after long turnover time of clinical specimens. BMC Infectious Diseases. Dis. 2014; 14, 636. 10.1186/s12879-014-0636-7 PMC4264541 25433390

[21] Ross BC, Johnson PD, Oppedisano F, Marino L, Sievers A, Stinear T. et al Detection of *Mycobacterium ulcerans* in environmental samples during an outbreak of ulcerative disease. Applied and Environmental Microbiology. 1997; 63: 4135–4138. 10.1128/AEM.63.10.4135-4138.1997 9327583 PMC168730

[22] Dorothy YM., Thomas B., Ernestina MQ., Samuel O., David OA., Gerd P. Evaluation of decontamination methods and growth media for primary isolation of *Mycobacterium ulcerans* from surgical specimens. Journal of Clinical Microbiology. 2004; 42: 5875–5876. 10.1128/JCM.42.12.5875-5876.2004 15583329 PMC535292

[23] Franzblau SG., Drug susceptibility testing of *Mycobacterium leprae* in the BACTEC 460 system. Journal of Antimicrobial Chemotherapy. 1989; 33: 2115–2117. 10.1128/aac.33.12.2115 PMC172831 2694952

[24] Zingue D, Panda A, Drancourt M. A protocol for culturing environmental strains of the Buruli ulcer agent, *Mycobacterium ulcerans*. Scientific Reports. 2018; 8: 6778 10.1038/s41598-018-25278-y 29712992 PMC5928104

